# A novel model for predicting the productivity index of horizontal/vertical wells based on Darcy's law, drainage radius, and flow convergence

**DOI:** 10.1016/j.heliyon.2024.e25073

**Published:** 2024-01-22

**Authors:** Roland I. Nwonodi

**Affiliations:** Federal University Wukari, Department of Chemical Engineering, Nigeria

**Keywords:** Drainage radius, Geometric pseudoskin factor, Flow convergence, Box-shaped or circular reservoir, Productivity index of horizontal or vertical wells, Darcy's law

## Abstract

The Productivity Index (PI) of oil wells is essential to developing a field's production plan, and previous studies report different models for predicting the PI of horizontal or vertical wells. However, predicting the PI of horizontal and vertical wells using a single cost-effective model remains unaccomplished. The models only predict results for either box-shaped or circular reservoirs. Thus, this study reports a new model for predicting the PI of horizontal and vertical wells in any reservoir type. The model incorporates the length of the horizontal section and formation thickness to Darcy's equation, and forms empirically the external drainage radius using vertical and horizontal radii. It includes both geometric pseudoskin (accounting for fluid convergence) and empirically determined correction parameters. The derived PI is validated using data from box-shaped and circular reservoirs, and the results showed that changes in permeability, well length, or well radius affect the PI for both reservoir types. In addition, the drainage radius of the horizontal well increased non-linearly with the PI. Sensitivity analyses of 20,000 runs and 20,000 trials, carried out with the Crystal Ball software, showed that the viscosity and formation volume factor were the most important variables that affected the PI, for both reservoir types.

## Nomenclature

∑s =: total skin in the well (−)
ΔP =: reservoir pressure minus well pressure (psi)
A =: cross-sectional area in Darcy's equation (sq. ft)
$A_{\text{drain}}$ =: drainage area (sq. ft)
$A_{e}$ =: elliptical drainage area (sq. ft)
$a_{H}$ =: width of the reservoir (ft)
$A_{hw}$ =: drainage area of the horizontal well (sq. ft)
$A_{r}$ =: radial area of flow through the well (sq. ft)
$A_{T}$ =: total drainage area of the reservoir (sq. ft)
$A_{vw}$ =: drainage area of the vertical
$A_{w}$ =: drainage area of the well (sq. ft)
$a^{\prime }$ =: major external radius of the well (ft)
$B_{H}$ =: breadth of the reservoir (ft)
$B_{o}$ =: oil formation volume factor (FVF) (RB/STB)
$b^{\prime }$ =: minor external radius of the well (ft)
β =: anisotropy factor of permeability (−)
$C_{H}$ =: shape factor (−)
dP =: pressure differential causing flow (psi)
dr =: differential radius (ft)
$d_{z}$ =: position of the well from the base of the vertical plane (ft)
$f_{f}$ =: dimensionless factor due to friction in Eq. (49) (−)
$f_{\text{ac}}$ =: dimensionless factor due to accumulation in Eq. (49) (−)
$f_{b}$ =: dimensionless factor due to pipe bend in Eq. (49) (−)
$h_{1}$, $h_{2}$ =: hydraulic heads a points 1 and 2 (ft)
h, H =: formation net thickness (ft)
$H_{r}$ =: sum of formation's thickness and wellbore length (ft)
J =: Productivity Index (PI) in the inflow equation (STB/D/psi)
$J_{w}$ =: PI of the wellbore (STB/D/psi)
$J_{h}$ =: PI of the horizontal well (STB/D/psi)
k =: unaltered permeability of the formation (md)
$k_{h}$ =: permeability in the y-direction ox horizontal permeability (md)
$k_{v}$ =: permeability in the vertical direction (md)
$k_{x}$ =: altered permeability in equation (24) (md)
$K_{x}$ =: permeability in the x-direction (md)
$K_{z}$ =: permeability in the z-direction (md)
L, $L_{w}$ =: wellbore length (ft)
$P_{R}$ =: reservoir pressure (psi)
$P_{\text{wf}}$ =: pressure of the flowing well (psi)
$P_{R}-P_{\text{wf}}$ =: pressure drawdown (psi)
$P_{D\;}$ =: dimensionless parameter related to the dimension of the reservoir (−)
q =: production rate from the well (STB/D)
q =: volumetric flow rate (STB/D)
$q_{h}$ =: flow rate in horizontal well (STB/D)
$q_{v}$ =: flow rate in vertical well (STB/D)
$q_{w}$ =: flow rate of the well (STB/D)
$q_{T}$ =: total flow rate in the reservoir (STB/D)
r =: radial distance from well circumference (ft)
$r_{w}$ =: well radius (ft)
$r_{w}^{\prime }$ =: corrected well radius (ft)
$r_{ev}$ =: drainage radius of the vertical section of the well (ft)
$r_{\text{eh}}$ =: drainage radius of the horizontal well (ft)
$r_{f}$ =: drainage radius factor (−)
$r_{x}$ =: radius of the damaged region (ft)
S =: skin factor (−)
$S_{c}$ =: skin due to convergence (−)
$S_{d}$ =: skin due to damage (−)
s_e_ =: eccentricity factor in the vertical direction (−)
$S_{g}$ =: geometric skin (−)
$S_{p}$ =: skin due to penetration (−)
μ =: viscosity of the fluid (cP)
$\mu_{o}$ =: oil viscosity (cp)
Z =: length of flow (ft)

## 1 Introduction

An essential parameter that oil companies use to develop a production plan for a field is the Productivity Index (PI) of oil wells, which measures the production potential. The production rate and pressure drawdown of an oil well are connected by the expression in Eq. (1), which is the inflow performance equation [[1], [2], [3], [4], [5]]:

(1) $$q=J_{w}\left(P_{R}-P_{\text{wf}}\right)$$

Thus, oil operators can derive the expression for the productivity index of oil wells using Eq. (2):

(2) $$J_{w}=\frac{q}{P_{R}-P_{\text{wf}}}$$

From Eq. (2), oil companies can calculate a well's PI using production or historical data. However, these historical data might be unavailable or insufficient, and the companies would still need to make economic (or technical) decisions. This is true, particularly in the unexplored or underdeveloped petroleum regions. The complexity of exploration is high [6] and stakeholders spend a lot of money developing new fields. These stakeholders may require an understanding of the production performance to assess the payback period of their initial investment. As a result, having a practical model that can produce precise results is crucial for making decisions quickly. However, one may derive the parameters of the model using the production data from the region to generate more accurate results.

In striving to supply the world's energy demands, oil companies have drilled both horizontal wells [[7], [8], [9], [10], [11]] and vertical wells [1,2,12], with varying production potentials, construction processes, and well costs. Vertical wells require a simple drilling technique to construct, while deviated wells are more technical and costlier to construct. In addition, the companies have evaluated the PI of these types of wells separately. However, the production potential of horizontal wells is greater than that of conventional non-horizontal wells [13]. Thus, horizontal wells are arguably the most sustainable and dependable means of supplying global oil demand. This sustained supply can help to maintain and improve global industrialization and keep the petroleum industry at a competitive advantage over its rivals.

**Fig. 1:**
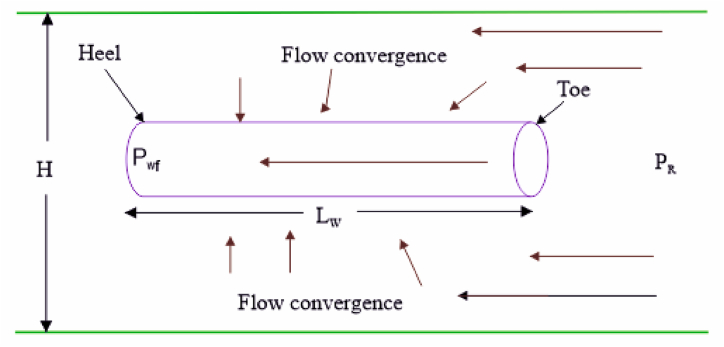
Well-reservoir flow physics from toe to heel.

Many scholars have studied the PI of horizontal wells [5,6,14] and vertical wells [1,2,12] over the years. The main components adopted in the studies are Darcy's equation, reservoir geometry, and the flow regime, which varies for vertical or horizontal wells. In addition, the major parameters applied are permeability (and its anisotropy), length of the horizontal section, formation thickness, pressure drawdown, fluid viscosity, well and external radiuses, well drainage, and skin factor. The flow regime, well's drainage radius, and skin factor are the most crucial factors that are utilised to distinguish the productivity of vertical wells from those of horizontal wells. The skin factor accounts for the damage caused by drilling, workover, or completion activities [15]. The flow regime and mechanisms of production in horizontal wells are more complex than in vertical wells [1]. In addition, this flow regime comprises both radial and linear flow. The simple assumptions applied in vertical wells (e.g., single permeability or radial symmetry) may not apply to a horizontal well [16,17]. This makes it difficult to estimate the PI of horizontal wells using the model for vertical wells. However, oil companies and other stakeholders may be curious to know whether a model developed for horizontal wells may apply to vertical ones. As the reservoir energy drives the fluids toward the well, the radial flow comes from all directions within the formation's thickness [1,10]. There might be an edge [5] or bottom water [17] in the aquifer. The linear flow comes from the movement of fluids through a non-changing cross-section, with both ends open to the flow and no fluids crossing the lateral or vertical sides [1,8]. During production, there may be fluid convergence, as Fig. 1 shows. This convergence causes additional pressure drop apart from the reservoir drawdown.

To evaluate the performance of a given well, an operator needs accurate knowledge of the drainage radius, apart from the well radius. Without the drainage radius, it is impossible to determine the PI of horizontal wells. In addition, the drainage radius of a horizontal well is larger than that of a vertical well [18]. Thus, one can infer that horizontal wells may have a lower pressure drop because of the pressure-volume relationship. The reservoir energy and fluids do not travel too far before contacting the well. Thus, under the same reservoir thickness, they produce more fluids than vertical wells. Although it may be easy to derive the drainage radius of vertical wells, the same is not true for horizontal wells. Joshi [7] presented two models for deriving the drainage radius of horizontal wells, which are the rectangular and elliptic models. However, the models yield different results. Yet, the elliptic model has formed the convention for defining well spacing for production purposes. Moreover, Abdelgawad and Malekzadeh [18] presented results that differ significantly from Joshi's elliptic model. The basis of their study was interference testing of flowing wells. From the reservoir-well geometry, one may predict representative values of the drainage radius for use in Darcy's equation.

The main area of concern is that it is rare to find an equation that can predict the PI of both types of wells in any reservoir type. This gap in research needs elimination. It is possible to do this since there is an associated vertical section to every horizontal well. Achieving this will yield a cost-effective model for the petroleum industry. In addition, it will be beneficial for operational use, since it is possible to convert a vertical well into a horizontal well [18]. However, it is curious to know how one can exploit Darcy's equation for such a purpose. It is important to know what parameters affect the outcome of the model or what form the derived model will take. That is whether it will take the form of the conventional model for vertical wells or those of horizontal models. It is interesting to know how to harness the flow regime, drainage radius, and convergence effect. Thus, this study aims to develop a novel model for estimating the PI of horizontal or vertical wells using Darcy's equation and Eq. (2). This will be an advantage over contemporary models. The objective is to include the conventional parameters for evaluating the PI of oil wells in the formulation. The targets are to develop the drainage radius of the horizontal well using the vertical section and include the pseudoskin due to convergence. The study strives to apply the model in a box-shaped reservoir.

Darcy's equation is the basis for the development of the flow rate through porous media. Darcy [19] experimented with the flow of water through sand packs and derived Eq. (3) for the relationship between the flow rate and the hydraulic heads:

(3) $$q=-\frac{\text{kA}}{\mu Z}\left(h_{2}-h_{1}\right)$$

The use of other liquids apart from water yields similar results. However, when applied to oil-producing wells, the most important representation of Darcy's equation is the following expression in Eq. (4):

(4) $$q=-\frac{\text{kA}}{\mu_{o}B_{o}}\frac{\text{dP}}{\text{dr}}$$

The oil FVF enters the equation because oil loses some mass of dissolved gases as it moves to the surface, during production. The dissolved gases are associated with the oil under reservoir conditions. The flow through the reservoir is mainly a complex flow of radial and linear dimensions.

Applying Darcy's equation for PI prediction requires a physical geometry. Babu and Odeh [20] presented a physical model of the production system used to predict the PI of horizontal wells, which is a box-shaped model, but Joshi [7], Abobakar et al. [12], and Amini and Blasingame [21] presented a circular reservoir. In the box-shaped model, the well is inside a well-defined reservoir with dimensions of thickness, length, and width. In addition, the well is located in a position to maximise the recovery from the reservoir. For the circular geometry, the well spacing defines the drainage area. However, the rock physics of Fig. 1 is the convention adopted in any of the models.

## 2 Literature review on related concepts

Many studies have attempted to develop models for the PI of horizontal wells. Among these are the conventional studies, which find a basis in Darcy's equation, for example, Joshi [7], Economides et al. [9], Babu and Odeh [20], Rennard and Dupuy [22], and Giger et al. [23]. The earliest approach used to derive models for the PI of horizontal wells was to assume that they were vertical wells lying on their sides [24]. By this assumption, the authors applied the equation for a vertical well to a horizontal one, for only the radial flow; however, the horizontal section of the well penetrates laterally into the reservoir. In addition, the large ratio of the well length to formation thickness means that complex flow is involved. Thus, there was a need to consider various boundary conditions. Subsequently, some authors considered the issues of skin effects due to damage, entry, non-Darcy flow, and unsteady pressure. Some workers considered the constant pressure drop along the pipe, for example, Economides et al. [9], Rennard and Dupuy [22], and Giger et al. [23], while others adopted the non-constant pressure drop from friction, acceleration, or gravity [25,26]. To advance the earliest concept, some workers assumed that the horizontal well comprised several vertical wells of differential lengths over the given extent of the reservoir [4]. Some scholars developed conventional models for the PI based on the complexities of both radial and linear flow in elliptical drainage. However, the models do not predict the exact relationship between the drainage of a horizontal well and a corresponding vertical one. The current study attempts to propose an exact relationship between horizontal drainage and vertical drainage. The conditions applied in the conventional models are steady-state or semi-steady state flow, geometric skin, well located in the reservoir to maximise inflow, constant pressure, and elliptic drainage.

Borisov carried out one of the pioneer studies on the development of the PI of the horizontal well in 1964 [27]. He assumed the elliptical drainage area, isotropic formation, and steady-state flow of reservoir fluids with constant boundary pressure. The mathematical expression developed for an incompressible fluid is the expression defined in Eq. (5):

(5) $$J_{h}=\frac{0.00708hK_{h}}{\mu B_{o}\left(\mathrm{Ln}\left(\frac{4r_{\text{eh}}}{L}\right)+\beta \frac{h}{L}\ln\frac{h}{2\pi r_{w}}\right)}$$

(6) $$\beta =\sqrt{\frac{K_{h}}{K_{v}}}$$

Note that Eq. (5) accounts for the anisotropy in permeability, as defined in Eq. (6). The author obtained the drainage radius of the horizontal well from the acre space or well spacing of the vertical well.

Giger et al. [23] predicted the PI for isotropic reservoirs with equal permeability in the vertical and lateral directions. They included the length of the well in the well's productivity rather than the product of permeability and thickness. The authors later improved on the model by incorporating the anisotropy in permeability and developed the following expressions in Eq. (7) and Eq. (8):

(7) $$J_{h}=\frac{0.00708LK_{h}}{\mu B_{o}\left(\frac{L}{h}\ln\;X+\beta \;\ln\frac{h}{2\pi r_{w}}\right)}$$

(8) $$X=\frac{2r_{\text{eh}}+2r_{\text{eh}}\sqrt{1+{\left(\frac{L}{2r_{\text{eh}}}\right)}^{2}}}{L}$$

Joshi proposed a new model to obtain the drainage area of the horizontal well and developed Eqs. (9), (10), (11) for the PI [28]:

(9) $$J_{h}=\frac{0.00708hK_{h}}{\mu B_{o}\left(\ln\;R+\frac{\beta h}{L}\ln\frac{\beta h}{2r_{w}}\right)}$$

(10) $$R=\frac{a+\sqrt{a^{2}-{\left(0.5L\right)}^{2}}}{0.5L}$$

(11) $$a=0.5L{\left(0.5+\sqrt{0.25+{\left(\frac{2}{L}r_{\text{eh}}\right)}^{4}}\right)}^{0.5}$$

The model is for the steady-state flow of slightly compressible fluid, single-phase flow, constant pressure at the well, and no damage.

Babu and Odeh [20] carried out a rigorous study to develop a solution to the diffusivity equation using a box-shaped reservoir. They used a non-constant well pressure and introduced the convention for the dimensions of the reservoir-well system. Their model considered a constant influx of slightly compressible fluid into the well with uniform completion. They also considered uniform damage along the well's path and assumed that the sides of the well align with the direction of the principal permeability. Using the no-flow boundary condition and pseudo-radial flow, they derived Eq. (12) for the productivity index and Eq. (13) for the flow area, which is the product of the reservoir thickness and reservoir breadth:

(12) $$J_{h}=\frac{0.00708B_{H}\sqrt{K_{h}\text{Kv}}}{\mu B_{o}\left(\ln\frac{\sqrt{A}}{r_{w}}+\ln\;C_{H}+S_{p}+\frac{s_{d}B_{H}}{L_{w}}-0.75\right)}$$

(13) $$A=a_{H}h$$

Rennard and Dupuy [22] developed an equation for the PI of horizontal wells, as presented in Eq. (14). The equation is similar to Joshi's model [7] but contains a corrected well radius (shown in Eq. (15)) and trigonometric function. The mathematical expression for the PI, without skin damage, is the following expression:

(14) $$J_{h}=\frac{0.00708hK_{h}}{\mu B_{o}\left(\mathrm{Cos}\;h^{-1}\left(\frac{2a}{L}\right)+\frac{\beta h}{L}\ln\frac{h}{2\pi r_{w}^{\prime }}\right)}$$

(15) $$r_{w}^{\prime }=\frac{\left(1+\beta \right)r_{w}}{2\beta }$$

It may be hard to find the inverse hyperbolic cosine of the values in the bracket, but the model applies to an elliptic, circular, or box-shaped reservoir.

Economides et al. [9] developed a method for estimating the PI of horizontal wells in box-shaped reservoirs. One can apply the method to multilateral wells in the same plane and the well need not align with the direction of the principal permeability. The mathematical expressions for calculating PI by this method are Eqs. (16), (17):

(16) J=k‾BH887.22Boμo(PD+BH∑s2πLw)

(17) $$P_{D}=\frac{B_{H}C_{H}}{4\pi h}+\frac{B_{H}S_{c}}{2\pi L_{w}}$$

As presented in the introduction section, there are two major methods used in estimating the drainage areas of horizontal wells, which include the elliptical drainage of Eq. (18). The elliptical drainage has both minor and major axes, which are defined by Eqs. (19), (20), (21) as follows [2,7]:

(18) $$A_{e}=\pi a^{\prime }b^{\prime }$$

(19) $$a^{\prime }=\frac{L}{2}+r_{\text{ev}}$$

(20) $$b^{\prime }=r_{\text{ev}}$$

(21) $$r_{\text{ev}}=\sqrt{\frac{43560*\text{acres}}{\pi }}$$

Several scholars have used this method to obtain the drainage radius of the horizontal well, but geometric approximations were employed in the process [22,27,28]. Some authors assumed that the reservoir is a rotating ellipsoid, with the ends of the horizontal well holding the focal points of the elliptic drainage [29]. It may be unphysical for the flow to be rotating, and there will be at least two rotating radii in the ellipsoid. Thus, the physical interpretation of this drainage radius may not be clear. Amini and Blasingame [21] showed the geometry of elliptical drainage using the flow into a fracture of a known length. The fracture half-length represented the length of the well in their model. This is merely a convenient way of dealing with the complex nature of the drainage of the horizontal well because one can consider the radiuses in different lateral directions. The second Joshi's method consists of two semicircles at the ends of the well, each with the external radius of the vertical section, and a rectangular section consisting of the well's length. Joshi [7] presented the expression of this geometry as follows in Eq. (22):

(22) $$A_{\text{drain}}=2b^{\prime }L+\pi b^{\prime 2}$$

Equation (22) resembles the equation of the surface area of a cylinder opened at only the ends; however, it inaccurately depicts the surface area. With these methods, it is difficult to have a well-defined relationship between the drainage radius of a horizontal well and a vertical one.

The study on fluid flow via porous media presented by Abdelgawad and Malekzadeh [18] differs from Joshi's elliptical drainage. They demonstrated that the length of a horizontal well correlated directly with the drainage area ratio of horizontal wells to vertical wells. Using the production rate ratio of horizontal wells to vertical wells, they presented a comparable trend. The drainage area ratio between horizontal and vertical wells was highest in the longest horizontal wells. A previous study on the drainage area of wells, carried out by Golan and Whitson [30], is consistent with their work. For wells produced from the same reservoir, the authors presented Eq. (23) as an approximation for the drainage area of a well:

(23) $$A_{w}=A_{T}\frac{q_{w}}{q_{T}}$$

The total skin around the circumference of the well affects PI because of changes in flow behaviour resulting from mechanical or non-mechanical factors. For example, when the flow converges into the well's perforations, it changes its flow behaviour. Thus, the skin factor is often incorporated into models for fluid flow to obtain more representative results for the PI. One of the fundamental studies on the skin factor traces to the work of Hawkins [31], who represented the skin as a region of impaired permeability and used Eq. (24) to relate the permeability to the radius of the well and that of the damaged region:

(24) $$S=\left(\frac{k}{k_{x}}-1\right)\ln\left(\frac{r_{x}}{r_{w}}\right)$$

Van-Everdingen [32] treated the skin as a region with reduced permeability in an infinitesimal thickness around the well. However, when applied to negative skin factors, the results were physically contradictory; thus, Hurst [15] introduced the concept of the effective well radius to enable dealing with both negative and positive skin.

The total skin in a well can comprise factors due to damage, partial completion, or partial penetration. Babu and Odey [20] defined this total skin in terms of damage and geometric skin as presented in Eq. (25):

(25) $$S_{t}=S_{g}+\frac{h}{L}S_{d}\sqrt{\frac{K_{h}}{K_{v}}}$$

The geometric skin results from the geometry of the flow as flow converges into the well and around the perforations. Thus, it can comprise the skin factors due to convergence and/or partial penetration, depending on the flow regime. Nevertheless, the skin factor due to convergence is active in radial, pseudo-radial [33], or late linear flow regimes.

Odey and Babu [16] presented Eq. (26) as the expression of the skin due to the convergence of fluids into the well:

(26) $$s_{c}=\left\langle \ln\frac{h}{r_{w}}+0.25\;\ln\frac{K_{x}}{K_{z}}-\ln\left(\sin\frac{\pi d_{z}}{h}\right)-1.838\right\rangle$$

This equation, developed mainly for a box-shaped reservoir, yields results similar to that of Kuchuk [8]. Economides et al. [9] also developed a model for the skin due to convergence for the PI using the well, reservoir, and an eccentricity factor as shown in Eq. (27) and Eq. (28):

(27) $$S_{c}=\ln\left(\frac{h}{2\pi r_{w}}\right)-\frac{h}{6L_{w}}+S_{e}$$

(28) $$S_{e}=\frac{h}{L_{w}}\left(\frac{2d_{z}}{h}-\frac{1}{2}{\left(\frac{2d_{z}}{h}\right)}^{2}-\frac{1}{2}\right)-\ln\left(\mathrm{Sin}\left(\frac{\pi d_{z}}{h}\right)\right)$$

The parameter $S_{e}$ equals zero when the well is in a central position in the vertical plane, and the value of the skin factor differs only slightly from that of Babu and Odeh [20].

## 3 Methodology

In this study, the author derived a model for predicting the PI of both horizontal and vertical wells based on Darcy's equation for flow through porous media. Appropriate boundary conditions (BCs) were applied and the geometric skin from the fluid convergence into the perforated well was added. The study provides a means to understand the effects of reservoir and well variables on the PI of box-shaped or circular reservoirs. In addition, it provides a cost-effective technique for evaluating the PI of vertical or horizontal wells. Subsequent research may focus on the more generally oriented well.

To achieve the novelty, the radial area in Darcy's equation was modified to contain a length scale, which comprised the length of the horizontal well and the formation thickness. This enabled the model to apply to a vertical or horizontal well. Conventional studies have argued that horizontal wells drain an elliptical volume [7,21,34]. This drainage volume seems to consist of elements from radial and linear length scales. Thus, the areal extent of the study covered both linear and radial dimensions to consider the well length and formation thickness. The author considered the external drainage radius as a displacement vector of the vertical and horizontal radii. Thus, a mathematical expression for the effective radius of the system was established. From empirical observations of previous studies, the areas drained by horizontal wells are larger than the vertical wells [18,28,30]. Thus, the author posed a proportional relationship between the area drained by a well and the well length. The application of this relationship to the horizontal well and its vertical section yielded a new expression between the drainage radius of the horizontal well and its vertical section. These components of radii were substituted into the resultant drainage radius of the system.

To obtain a closed-form solution for the PI, a geometric pseudoskin factor was added. This pseudoskin factor was derived from the convergence of fluid around the circumference of the horizontal well. The fluid convergence resulted from the opening of the well's perforations, causing fluids to move in the vertical direction. In addition, provision was made for permeability anisotropy. To be consistent with the goals of this study, the geometric skin in a vertical well differs from that in a horizontal well. So, the geometric skin was presented as a natural logarithm function of the ratio of the sum of well length and formation thickness divided by the formation thickness, to make the effect of the geometric skin negligible in a vertical well. The substitution of the drainage radius and the geometric skin into the derived flow equation yielded the model for the PI, based on Eq. (2). The derived model was tested using data from Well A and Well B in box-shaped and circular reservoirs, respectively. See Table 1 and Table 2 for the properties of the wells.

**Table 1:** Characteristics of Well A used for model testing.

Parameter	Value	Parameter	Value (ft)
Drainage geometry	Box-shaped	$D_{x}$	1800
$K_{v}$	18 md	$D_{y}$	1800
$K_{h}$	90 md	$D_{z}$	150
μ	1.1 cp	$a_{H}$	1800
$B_{O}$	1.21 RB/STB	$b_{H}$	3800
H	150 ft	$d_{z}$	50
$L_{W}$	1300 ft	$d_{y}$	900
$d_{x}$	900 ft	$r_{w}$	0.27

Use the conversion factors: I ft = 0.3048 m. 1 cp = 0.001 Nsm^−2^. 1 psi = 6895 Pas. 1 acre = 4046.856 m^2^. 1 md = 9.869E-16 m^2^. 1 STB/D/psi = 2.305 E−5 m^3^/d/pas.

**Table 2:** Well B in circular reservoir data used for model validation [1].

Parameter	Value	Parameter	Value
Drainage area	120 Acres	Formation thickness	60 ft
Vertical permeability	100 md	Well length	2000 ft
Horizontal permeability	100 md	Well radius	0.3 ft
Viscosity	0.9 cp	Formation volume factor	1.2 RB/STB
Reservoir Pressure	3000 psi	Well pressure	2500 psi

**Fig. 2:**
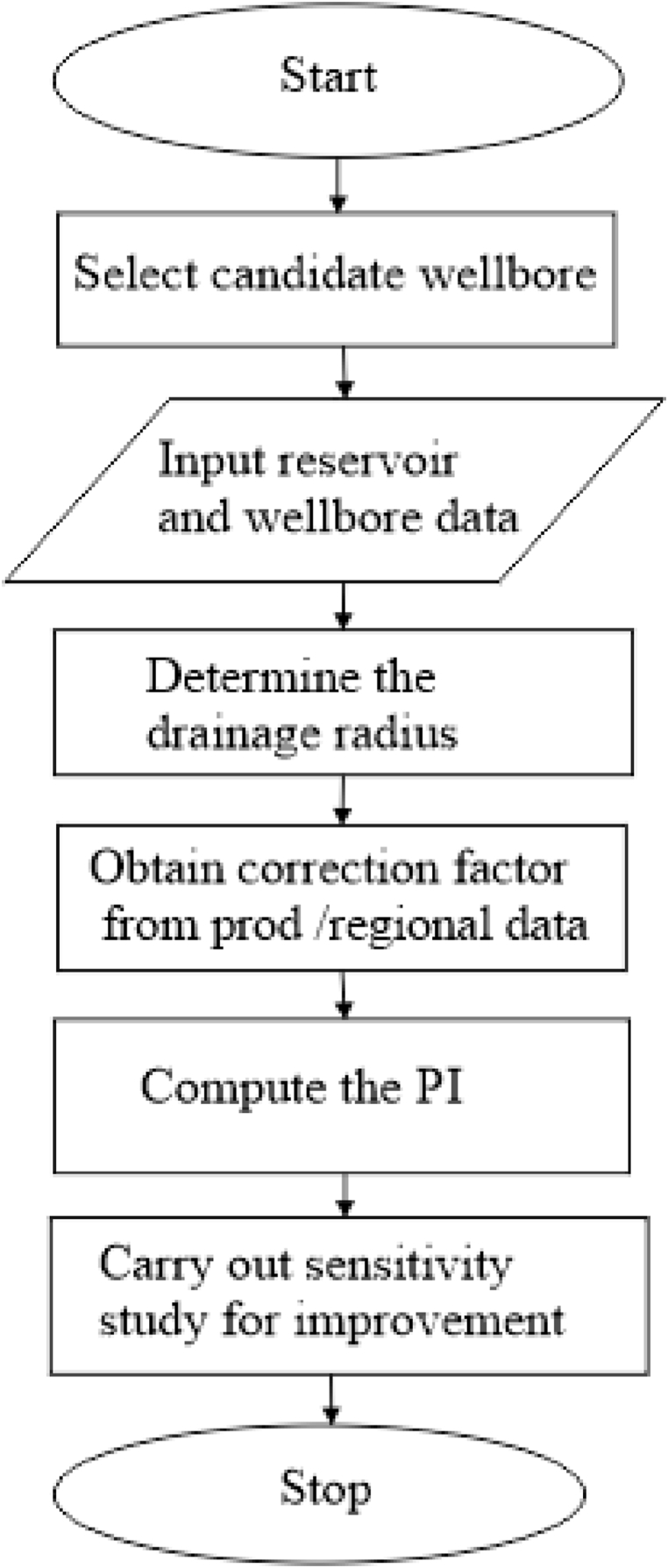
The flow chart for the technical route of the study.

The model parameter of Well B was derived using a pressure test model obtained from the late pseudo radial flow [33]. Other flow regimes may be adopted for future studies. Well-defined simulations were run on the box-shaped and circular reservoirs using the Crystal Ball software, based on the Monte Carlo sampling. The aim was to consider the sensitivity analysis for both wells. The process variables selected for the analysis were 20,000 runs under 20,000 trials and a 95 % confidence level. The assumptions applied for both reservoir types were not the same. The assumptions applied were the FVF, viscosity, well length, formation thickness, horizontal permeability, vertical permeability, and well radius. The acre spacing was included for the circular reservoir while parameters related to well position were added for the box-shaped reservoir. Fig. 2 is the flow chart of the technical route of the study.

## 4 Theory and model development

The pressure drawdown is the energy available for the flow of reservoir fluids through the porous rock. This flow enters the well as a complex geometry that is mainly radial and linear based on permeability anisotropy and flow channel. The combination of the reservoir drive and open perforations causes flow convergence to the vertical and lateral directions of the well. Thus, the flow of hydrocarbon covers an area extent comprising the thickness of the formation and length of the well. The following premises are the basis for the development of this study.

1. There is mainly steady-state flow in the reservoir, which is controlled by Darcy's law.
2. Radial flow is significant in the reservoir and linear flow is active.
3. The horizontal well can produce over the entire section of its horizontal length.
4. The pseudo skin depends on well geometry, permeability, and formation thickness.
5. Fluid flow is homogeneous in properties but there can be permeability anisotropy.
6. The vertical and horizontal permeability controls the permeability anisotropy.
7. The well's position in the reservoir is aligned to maximise flow.
8. Fluid converges to the well over the entire thickness of the formation.
9. Frictional pressure drop at entry points is negligible.

Premises 1 to 4 allow the use of Darcy's law, which for the flow of oil in the porous rock is the following expression:

(29) $$q_{r}=\frac{A_{r}K}{\mu B_{o}}\frac{\text{dP}}{\text{dr}}$$

The radial area available for fluid flow is the following expression for the curved surface area:

(30) $$A_{r}=2\pi rH_{r}$$

This helps to include the well length and considers the flow over the entire converging region. Incorporating Eq. (30) into Eq. (29) yields the following expression in Eq. (31):

(31) $$q_{r}\frac{1}{r}\text{dr}=\frac{2\pi K_{h}H_{r}}{\mu B_{o}}\text{dP}$$

Naturally, the reservoir energy may drain oil from the sides or bottom of the well if there is edge water or bottom water aquifer, respectively [5]. Thus, the integral of the drainage radius is from the well to the external drainage of the reservoir, while that of the pressure drawdown is from the flowing well pressure to the reservoir pressure. This helps to cover the boundary over which the well may drain hydrocarbons. Thus, the following expression emerges for the system:

(32) $$q_{r}\int_{r_{w}}^{r_{e}}\frac{1}{r}\text{dr}=\frac{2\pi K_{h}H_{r}}{\mu_{o}B_{o}}\int_{P_{w}}^{P_{r}}\text{dP}$$

Evaluating the integrals in Eq. (32) yields the following expression:

(33) $$q_{r}\left(\ln\;r_{e}-\ln\;r_{w}\right)=\frac{2\pi K_{h}H_{r}}{\mu_{o}B_{o}}\Delta P$$

From Eq. (2), the representative model for the PI of the system becomes the following expression in Eq. (34):

(34) $$J_{w}=\frac{2\pi K_{h}H_{r}}{\mu_{o}B_{o}\left(\ln\;r_{e}-\ln\;r_{w}+S_{g}\right)}\Delta P$$

The external radius in Eq. (33) is the resultant external radius for the system. Thus, one can treat the radius as the following displacement vector:

(35) $$r_{e}=\sqrt{r_{\text{ev}}^{2}+r_{\text{eh}}^{2}}$$

Equation (35) indicates that the external radius for a vertical well is different from that of a horizontal well since their flow regime is different.

One can use the drainage of the vertical section to define that of the horizontal section since the wells drain more fluids from the reservoir as its length increases [18]. Then the following mathematical proportion derives under the same permeability and drawdown:

(36) $$\frac{A_{\text{hw}}}{A_{\text{vw}}}\propto L_{w}$$

The replacement of the proportionality in Eq. (36) with the formation thickness as the scaled-boundary condition yields the following expression:

(37) $$r_{\text{eh}}=r_{\text{ev}}\sqrt{r_{f}\frac{L_{w}}{h}}$$

The radius factor in the square root may be derived empirically or geometrically. Substituting Eq. (37) into Eq. (33) yields the following expression for the resultant external radius of the well:

(38) $$r_{e}=r_{\text{ev}}\sqrt{1+r_{f}\frac{L_{w}}{h}}$$

From the well spacing of vertical wells in a circular reservoir, one can obtain the drainage radius in Eq. (39) using the equation of a circle:

(39) $$r_{\text{ev}}=\sqrt{\frac{43560\times \text{Acres}}{h}}$$

It is assumed that the well is at the centre of the reservoir to harness the energy of the reservoir efficiently. For a box-shaped reservoir, the volume covers the product of the thickness, length, and width of the reservoir. Using the equivalent cylindrical volume, one may estimate the external radius of the vertical section of the box-shaped reservoir as Eq. (40):

(40) $$r_{\text{ev}}=\sqrt{a_{H}\frac{b_{h}}{\pi }}$$

Premise (4) allows the pseudoskin to apply to circular or box-shaped reservoirs. This pseudoskin considers the lumped effect of aspects not considered in the derivation of the PI, which includes radial and linear geometry effects. The geometric skin in this study accounts for the convergence of fluids into the well from its toe to heel. The closure of the perforations maintains a column of fluid along the horizontal direction, within the volume of the reservoir. This forms uniform streamlines. On open perforations, the streamlines, path lines, and streak lines all converge towards the perforations, and this leads to an increased pressure drop as the fluids strain themselves in the process. The ratio of the sum of the formation thickness and length of the well divided by the formation thickness affects the radial and linear convergence in the given volume. Thus, the author poses the following expression in Eq. (41) for the effective geometric skin:

(41) $$S_{g}=\ln\frac{h+L_{w}}{L_{w}}+S_{c}\sqrt{\frac{K_{h}}{K_{v}}}$$

The dimensionless ratio is inside the natural logarithm to make the effect of the convergence negligible in a vertical well.

The skin due to convergence relates to the anisotropy in permeability [8,9] and the dimension of the well [20]. This convergence skin covers a differential distance around the circumference of the well, which forms a dimensionless quantity when divided by radial distance. This quantity acts like a pseudo strain introduced as the fluids converge in the vertical direction along the horizontal section. Thus, the elemental skin is approximately equal to the strain from the convergence as Eq. (42) shows:

(42) $$R\frac{\text{dr}}{r}=\text{dS}$$

Since the skin around the circumference of the wells may vary with the permeability anisotropy, one can pose the integral of Eq. (42) as the expression in Eq. (43):

(43) $$R\int_{2\pi \beta r_{w}}^{2\left(1+\beta \right)\pi h}\frac{\text{dr}}{r}=\int_{-0.5S_{c}}^{0.5S_{c}}\text{dS}$$

NB: the integral in Eq. (43) is from half the negative skin to half the positive skin factor to cover the entire axis of the well. Therefore, the convergence skin for the horizontal well in this study is the following expression highlighted in Eq. (44):

(44) $$S_{c}=\text{Rln}\left(\frac{h\left(1+\beta \right)}{\beta r_{w}}\right)$$

The substitution of Eq. (38) and Eq. (41) into Eq. (34) yields Eq. (45) as the model for the PI (in field units):

(45) $$J_{w}=\frac{0.00708K_{h}\left(H+L_{w}\right)}{\mu_{o}B_{o}\left(\ln\frac{r_{e}}{r_{w}}+\ln\frac{H+L_{w}}{H}+\text{Rln}\frac{\left(1+\beta \right)h}{\beta r_{w}}\sqrt{\frac{K_{h}}{K_{v}}}\right)}$$

One can derive the parameter $r_{f\;}$ in Eq. (37) or (38) using either data from field production or the dimensions of the reservoir/well system. Without the availability of historical data, the combination of Eq. (35) and Eq. (38) yields the following expression in Eq. (46):

(46) $$r_{f}=\frac{H}{L_{w}}\left(\frac{r_{\text{ehx}}^{2}+r_{\text{ehy}}^{2}}{r_{\text{ev}}^{2}}\right)$$

## 5 Results and discussion

The model of this study applies to both box-shaped and circular reservoirs, depending on the value of the parameter in Eq. (45). From the data in Table 1, the PI obtained using Babu and Odeh's model [20] is 26.238 STB/D/psi, while the value obtained using Economides et al. [9] is 27.617 STB/D/psi (with ${\;b}_{H}=2a_{H},C_{H}=2.678)$. From Eq. (43), the drainage radius of the vertical section is 1475.25 ft, while Eq. (46) yields a value of 0.2318 for the parameter, $r_{f}$. (46). Therefore, the following becomes the value of the PI, based on the developed model and the value of unity for the parameter, R:

$$J_{w}=\frac{0.00708\times 90\times \left(150+1300\right)}{1.1\times 1.21\times \left(\ln\left(\frac{2559.22}{0.27}\right)+\ln\frac{150+1300}{150}+2.236\times \ln\left(\frac{\left(1+2.236\right)\times 150}{2.236\times 0.27}\right)\right)}=26.31\;\text{STB}/D/\text{psi}$$

The value obtained is between those of [9,20], but closer to Ref. [20]. Perhaps, this indicates an accurately chosen value for the parameter, R. Allowing the reservoir to flow with an isotropic permeability of 90 md yields an increase in the PI for all the models. The model by Babu and Odeh [20] yields 36.39 STB/D/psi, the one by Economides et al. [9] yields 38.41 STB/D/psi, while the study model yields 37.647 STB/D/psi. With the vertical permeability reduced to 5 md, Table 3 shows the results of the PI for the three models.

**Table 3:** Values of PI for Well A with vertical permeability reduced to 5 md.

Models	PI (STB/D/psi)
Babu and Odey [20]	17.697
Economides et al. [9]	18.975
Current study	17.737

The model performs reasonably well when one varies the other properties of the system. For example, Table 4 shows the results of the PI when the well radius changes to 0.5 ft.

**Table 4:** Values of PI for Well A with a radius of 0.5 ft.

Models	PI (STB/D/psi)
Babu and Odey [20]	27.857
Economides et al. [9]	29.414
Current study	28.461

The change in the value of the formation thickness showed a corresponding change in the PI of the models. With a value of 300 ft for the thickness, the PI increases as indicated in Table 5.

**Table 5:** Values of PI for Well A with a formation thickness of 300 ft.

Models	PI (STB/D/psi)
Babu and Odey [20]	30.720
Economides et al. [9]	32.428
Current study	28.01

The increase in the length of the well also indicated an increase in the PI of all the models, with the study model yielding an intermediate value. Table 6 shows the result when the length of the well increases to 2000 ft.

**Table 6:** Values of PI for Well A for a well length of 2000 ft.

Models	PI (STB/D/psi)
Babu and Odey [20]	42.248
Economides et al. [9]	33.214
Current study	38.427

To apply the developed model to circular reservoirs, consider Table 2 for the data of Well B. The parameter related to convergence skin is obtained as 3.517 using the pressure test model from late pseudo radial flow [33] and the data of the given well. From Eq. (45), the following value obtains:

$$J_{w}=\frac{0.00708\times 100\times \left(60+2000\right)}{0.9\times 1.21\times \left(\ln\left(\frac{2266.4}{0.3}\right)+\ln\frac{2000+60}{60}+3.517\times 5.99\sqrt{\frac{100}{100}}\right)}=40.266\;\text{STB}/D/\text{psi}$$

Table 7 shows the results obtained using the developed model and the conventional models applicable to circular reservoirs. These results indicate that the developed model can handle both circular reservoirs (when combined with history matching) and box-shaped reservoirs.

**Table 7:** Values of PI for Well B obtained using different conventional models.

Models	PI (STB/D/psi)
Joshi [7]	35.868
Borisov [27]	37.408
Geiger et al. [23]	46.390
Renard and Dupuy [22]	37.023
Current study	40.266

The permeability of the reservoir is a critical parameter that controls the flow of fluids to the well. However, the change in the vertical permeability accounts for a small change in the PI of Well B, compared to the change in the horizontal permeability. For example, the increase in the vertical permeability from 0 to 100 md caused the PI to increase from 31.306 to 40.2667 STB/D/psi, while the same increase in the horizontal permeability caused the PI to increase from 0 to 40.2667 STB/D/psi. Maybe the explanation is that, within the same range of permeability, there is more resistance to fluid seepage in the vertical direction than in the horizontal. Increasing the horizontal permeability of Well B to 200 md causes the PI of all the models to increase, as Table 8 shows. Hence, the companies should aim to increase the horizontal permeability to improve oil recovery. However, when permeability anisotropy increases, the PI of Well B reduces, according to Eq. (45). This is consistent with the studies of Abobakar et al. [12] and Yahui et al. [35].

**Table 8:** PI of Well B using different conventional models for 200 md.

Models	PI (STB/D/psi)
Joshi [7]	67.320
Borisov [27]	71.877
Geiger et al. [23]	82.660
Renard and Dupuy [22]	70.736
Current study	76.578

Practically, to obtain the PI of any horizontal well using the developed model, apply offset data from the field or reservoir where the well is located. For unexplored basins, use data from the geologic basin with the closest characteristics as a guild. We can use different flow regimes to derive the model parameter apart from the late pseudoradial flow. One benefit of the resulting model is its ability to forecast valuable and scientifically accurate outcomes. However, the outcomes ought to be restricted to the flow regime from which the model parameter was derived. The well does not have to be in a vertical location for the results to be valid for both homogeneous and heterogeneous reservoirs. It is unnecessary to substitute the equivalent permeability for the horizontal permeability as Giger and other scholars did for the heterogeneous reservoir [23]. For a well that might be producing from bottom water or an edge, the model can offer precise results.

Technically, the drainage radius shows the distance the reservoir energy travels as it pushes fluids into the well. Hence, a vertical well seems to take more time to experience the reservoir's drive. During this time, the energy of the reservoir experiences some dissipation. A horizontal well experiences the reservoir's drive much faster as fluids are being drawn from the reservoir. In addition, the reservoir does not lose most of its energy before the fluids enter the well. This supports the faster and larger recovery of oil in horizontal wells and highlights the importance of the accurate estimation of the drainage radius. The drainage radius developed in this study is consistent with the results obtained by Abdelgawad and Malekzadeh [18]. The model shows that the drainage radius of a horizontal well relates to that of the vertical by the factor inside the square root of Eq. (37). Thus, as the formation thickness reduces and the well length increases, the radius of the horizontal well becomes larger than that of the vertical well. To further support the validity of Eq. (37), one can exploit the drainage area in the work of Golan and Whitson [30]. When applied separately to a horizontal well and its vertical counterpart under the same total flow rate and reservoir area, one obtains Eq. (47):

(47) $$r_{eh}=r_{ev}\sqrt{\frac{q_{h}}{q_{v}}}$$

**Fig. 3:**
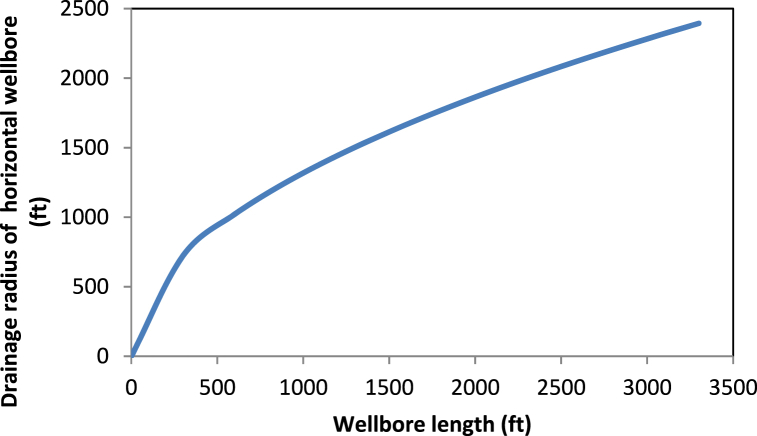
Variation of horizontal well's drainage radius with its length.

Allowing the production rates of the horizontal and vertical wells to be proportional to the well length and formation thickness, respectively, yields the exact form of Eq. (37). With the known external radius of the vertical section, it becomes possible to consider the variation of the drainage radius of the horizontal well with its lateral length or formation thickness. Fig. 3 shows the variation of the drainage radius of Well B with the well length according to Eq. (37), for constant formation thickness. When the well length equals zero, the external radius is equivalent to that of the vertical well, and the corresponding PI is for the vertical well. The drainage radius of the horizontal well dramatically increases with the length of the well. Therefore, we expect longer wells to have larger drainage radiuses under the same formation thickness. One can also compare the drainage radius of two different horizontal wells when data for their thickness, drainage area of the vertical radius, and length of the well are available.

**Fig. 4:**
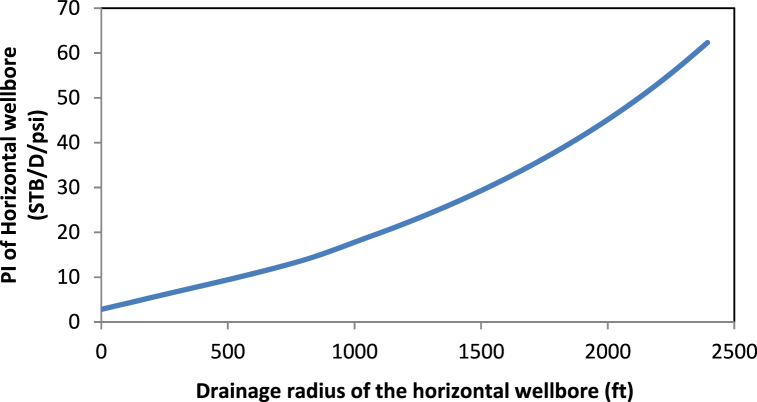
Variation of well length and drainage radius on PI of the horizontal well.

The larger the drainage radius of the horizontal well, the bigger the hydrocarbons produced from the reservoir as Fig. 4 shows. There will be a reduction in pressure drop as the reservoir has more energy to carry the fluids into the well.

**Fig. 5:**
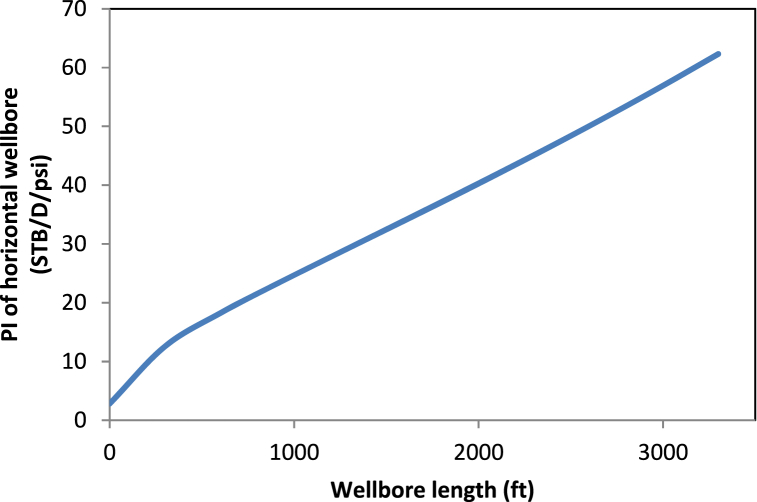
Variation of well length and drainage radius on PI of the horizontal well.

Thus, the PI increases with the drainage radius and well length, as indicated in Fig. 5.

As the length increased from zero, the PI also increased significantly. This trend is consistent with the results of Well A and those of previous studies [5,7,12,18,23,34]. Thus, a horizontal well that penetrates more into the productive zone will yield higher recoveries.

However, none of the previous models considered in this study has the sophistication to predict results for vertical wells as well as horizontal wells. This is an advantage of the current study. When the well length approaches zero, Eq. (45) approaches Eq. (48), which is for a vertical well with its total skin factor:

(48) $$J_{w}=\frac{0.0708\text{KH}}{\mu_{o}B_{o}\left(\ln\frac{r_{e}}{r_{w}}+S_{t}\right)}$$

From the data in tables 1 and 3, one finds that the PI of a vertical well can be lower than that of a corresponding horizontal well by up to five times. To obtain the downstream value of the PI, one needs to add the additional pressure drop from accumulation, friction, or possibly pipe bends. Then, Eq. (45) may be updated as Eq. (49):

(49) $$J_{w}=\frac{0.00708K_{h}\left(H+L_{w}\right)}{\mu_{o}B_{o}\left(\ln\frac{r_{e}}{r_{w}}+\ln\frac{H+L_{w}}{H}+RS_{c}\sqrt{\frac{K_{h}}{K_{v}}}+f_{f}+f_{\text{ac}}+f_{b}\right)}$$

**Fig. 6:**
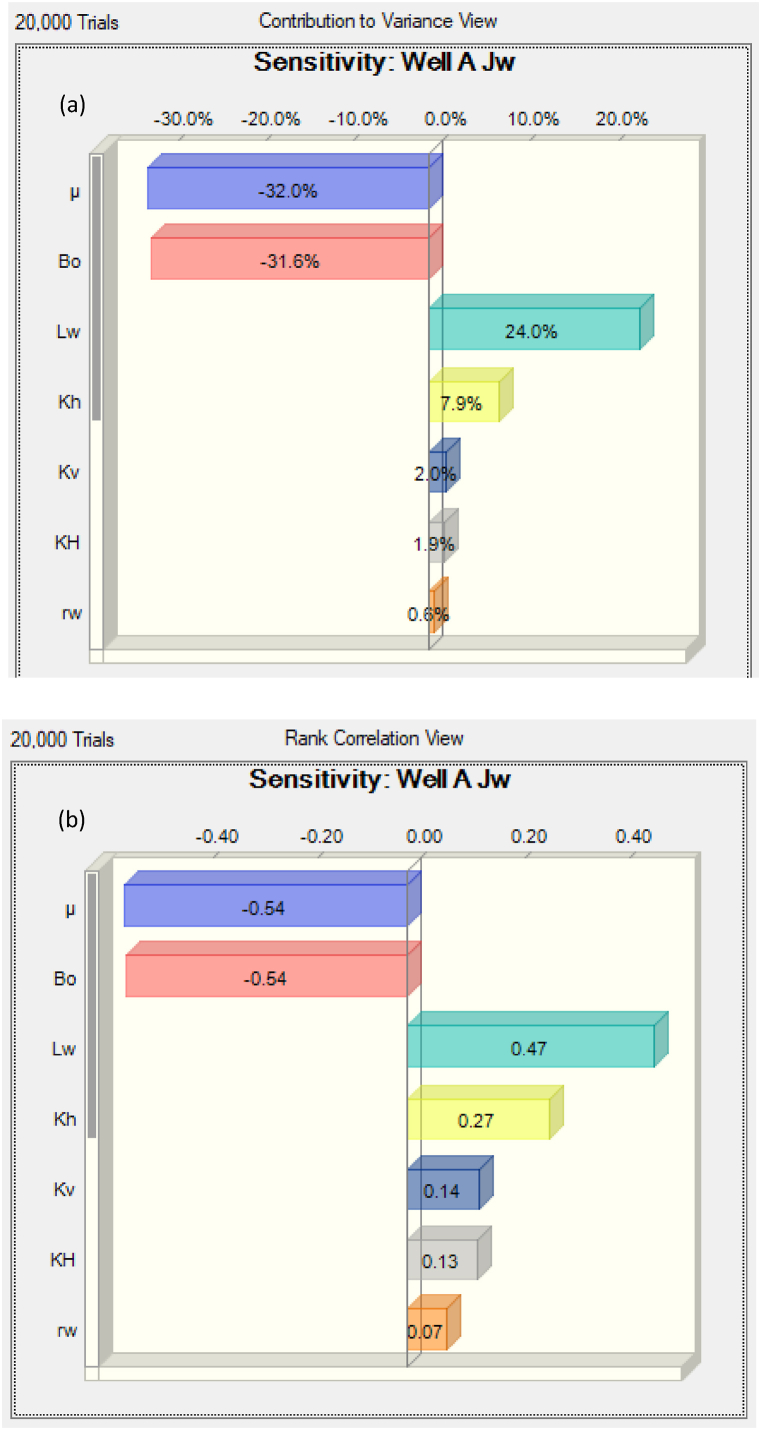
Sensitivity analysis of the variables affecting the PI of Well A. (a): Contribution to variance of the variables. (b): Rank correlation of the variables.

**Fig. 7:**
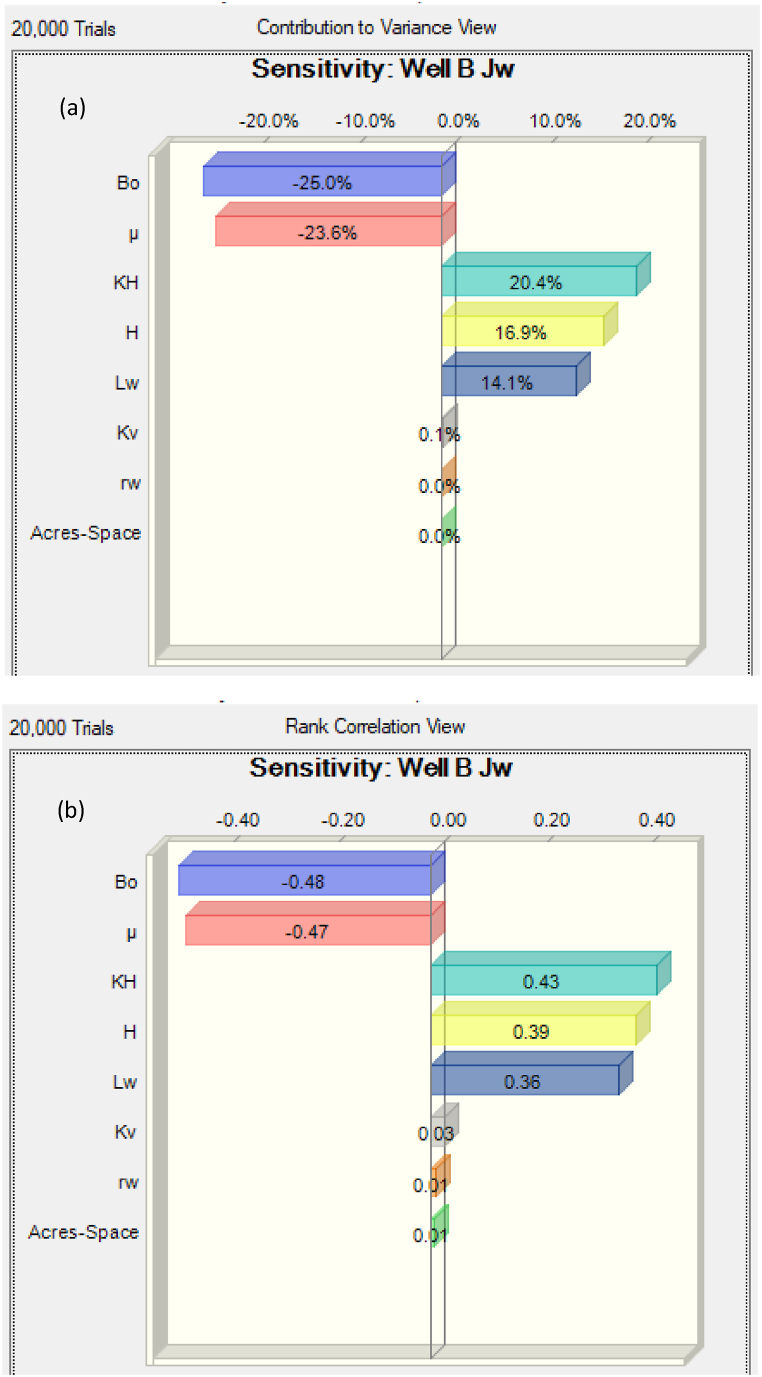
Sensitivity analysis of the variables affecting the PI of Well B. (a): Contribution to variance of the variables. (b): Rank correlation of the variables.

The next focus is on the sensitivity analyses of the variables affecting the PI of the two wells, using Crystal Ball. Fig. 6 shows the results for Well A (Box-shaped reservoir), while Fig. 7 shows that for Well B (circular reservoir).

For Well A, the results indicate that the most important uncertainties affecting the PI are those associated with the oil viscosity, oil FVF, well length, and horizontal permeability. The effects of the oil's FVF and viscosity were almost equal. The operators must determine the values of the variables accurately for any production system. The results showed that the oil FVF, oil viscosity, formation length, and well offset (in the x and z directions) are negatively correlated to the PI, while the other variables were positively correlated to the PI. See the summary of the rank correlation and contribution to variance presented in Table 9. Thus, it is clear that to optimize the performance of the box-shaped reservoir, oil companies should focus on reducing the oil FVF and oil viscosity, while increasing the well length and permeability in the lateral and vertical directions. To reduce the viscosity, the oil company can apply heated steam and CO_2_, as was done for the improvement of the performance of heavy oil flow through a horizontal well [11].

**Table 9:** Variance and Spearman's correlation obtained from the analysis for Well A.

Variables	Contribution to variance	Rank correlation
μ	32.0 %	−0.54
$B_{o}$	31.6 %	−0.54
$L_{w}$	24.0 %	0.47
$K_{h}$	7.9 %	0.27
$K_{v}$	2.0 %	0.14
$K_{H}$	1.9 %	0.13
$r_{w}$	0.6 %	0.07
H	0.1 %	0.02
$a_{H}$	0 %	−0.01
$d_{x}$	0 %	−0.01
$d_{z}$	0 %	−0.01
$d_{y}$	0 %	0.00
$b_{H}$	0 %	0.00

For Well B, the results indicate that the most important uncertainties affecting the PI are the oil FVF, oil viscosity, horizontal permeability, formation thickness, and well length. Other variables (acre spacing, well radius, and vertical permeability) had minimal effects on the variance. Thus, it is important to determine accurately the factors that affect the PI the most. The performance of the circular reservoir was only different from that of the box-shaped one because the horizontal permeability showed values higher than the effects of the well length and the formation thickness. Perhaps, this may stem from the difference in the reservoir geometry assumed. The oil FVF, oil viscosity, formation length, and well offset are negatively correlated to the PI, while the other variables show a positive correlation as Table 10 indicates.

**Table 10:** Variance and Spearman's correlation obtained from the analysis for Well B.

Variables	Contribution to variance	Rank correlation
$B_{o}$	25.0 %	−0.48
μ	23.6 %	−0.47
Acres-Space	0.0 %	0.01
$r_{w}$	0.0 %	0.01
$K_{v}$	0.1 %	0.03
$L_{w}$	14.1 %	0.36
H	16.9 %	0.39
$K_{H}$	20.4 %	0.43

Thus, oil companies should aim to improve the horizontal permeability of the circular reservoir, using hydraulic fracturing, for example, to improve the performance of oil wells. The application of heated steam and CO_2_ can further improve performance.

For both wells considered, the effect of the oil FVF is significantly high. Oil companies can exploit this to improve the PI by looking for techniques to reduce the value of oil FVF. Studies showed that the FVF of saturated oil is a function of temperature, the specific gravity of the fluids, and the solution gas-oil-ratio (GOR), with the latest having the most significant effect [36,37]. There is no control on the GOR except to exploit the pressure system of the well. Any well design that can maintain the oil FVF at approximately equal to unity is good for improving oil performance. If possible, oil companies should target well designs that can limit surface or receiving pressure to atmospheric pressure and average global temperature (that depends on geologic evidence).

## 6 Conclusions

This study presented a novel model (that is simple and cost-effective to use) for calculating the PI of both horizontal and vertical wells and showed details of how to improve oil well performance. It exploited Darcy's flow model for porous rocks, and derived equations for the external drainage and geometric skin due to fluid convergence. The novel model may be applied to box-shaped and circular reservoirs as long as model parameters are derivable. The results demonstrated the following points.

1. The major difficulty in estimating the PI of horizontal wells arises from the difficulty in defining correctly the well's drainage radius.
2. Physically, the drainage radius indicates the distance to available energy for recovery.
3. The function of the well length is to reduce the distance to the reservoir's energy; thus, it increases the available energy for production.
4. The drainage radius of the horizontal well correlates to that of the vertical well nonlinearly.
5. The reservoir geometry determines the sensitivity of the model parameters to the PI; however, the most significant factors are the fluid viscosity, oil FVF, horizontal permeability, and well length.
6. The productivity index of horizontal wells is more sensitive to horizontal permeability than the vertical permeability for both box-shaped and circular reservoirs.

## Data available statement

Data will be made available on request.

## CRediT authorship contribution statement

**Roland I. Nwonodi:** Writing – review & editing, Writing – original draft, Validation, Resources, Project administration, Methodology, Investigation, Formal analysis, Data curation, Conceptualization.

## Declaration of competing interest

The author declares that there is no known competing financial interests or personal relationships that could have appeared to influence the work reported in this paper.

## References

[1] Ahmed T. (2001).

[2] Chaudhri A.M. (2003).

[3] Knut-Andrea L. (2014).

[4] Liu C., Zhang J.T., Wu H.J., Zhang B., Wang L. (2018). Study and application of horizontal well productivity formula with continuous time-varying consideration. J. Power Energy Eng..

[5] Yuan H., Li W., Yuan Y., Luo J., Yan W. (2021). Productivity evaluation of horizontal wells in heterogeneous reservoirs with composite water aquifers. Journal of Petroleum Exploration and Production.

[6] Sun F.R., Yao Y.D., Li X.F., Yu P.L., Ding G.Y., Zou M. (2017). The flow and heat transfer characteristics of superheated steam in offshore wells and analysis of superheated steam performance. Comput. Chem. Eng..

[7] Joshi S.D. (1991).

[8] Kuchuk F.J. (1995). Well testing and interpretation of horizontal wells. J. Petrol. Technol..

[9] Economides M.J., Brand C.W., Frick T.P. (1996). Well Configuration in anisotropic reservoir. SPE Form. Eval..

[10] Ahmed T., Meehan D.N. (2011).

[11] Sun F.R., Yao Y.D., Li X.F., Li G., Han S., Liu Q., Liu W. (2018). Type curve analysis of the multi-phase flow of multi-component thermal fluid in toe-point injection horizontal wells considering phase change. J. Petrol. Sci. Eng..

[12] Abobakar E., Elsanoose A., Khan F., Rahman M.A., Aborig A., Noah K. (2021). Quantifying the partial penetration skin factor for evaluating the completion efficiency of vertical wells. J. Pet. Explor. Prod. Technol..

[13] Akangbou H.N., Burby N., Nasr G. (2017). Effectively optimizing the production of horizontal wells in homogeneous oil reservoirs. J. Petrol. Sci. Eng..

[14] Su Y.C., Shi H.F., He Y.F. (2018). A new method to calculate sweep efficiency of horizontal wells in heterogeneous reservoirs. J. Pet. Explor. Prod. Technol..

[15] Hurst W., Clark J.D., Brauer E.B. (1969). The skin effect in producing wells. J. Petrol. Technol..

[16] Odeh A.S., Babu D.K. (1990). Transient flow behaviour of horizontal wells: pressure drawdown and buildup analysis. SPE Form. Eval..

[17] Kumar M., Sharma P., Gupta D.K. (2017). Sensitivity study of horizontal length, offset from Water oil contact, and withdrawal rate of horizontal well in bottom water drive reservoir. J. Pet. Explor. Prod. Technol..

[18] Abdelgawad A., Malekzadeh D. (2001). Determination of the drainage area of horizontal wells in the presence of vertical wells: effect of reservoir and well parameters. J. Can. Petrol. Technol..

[19] Darcy H. (1856).

[20] Babu D.K., Odeh A.S. (1989). Productivity of a horizontal well. SPE Reservoir Eng..

[21] Amini S.I.D., Blasingame T.A. (2007). SPE Hyd. Frac. Tec. Conf. Texas.

[22] Rennard G., Dupuy J.M. (1990). Formation Dam. Control Symp..

[23] Giger F., Reiss L.H., Jourdan A. (1984). SPE Annual Technical Conference and Exhibition.

[24] Anklam E.G. (2001).

[25] Ozkan E., Sarica C., Haciislamoglu M., Raghavan R. (1993). Production Operations Symposium.

[26] Asheim H., Kolnes J., Oudeman P. (1992). A flow resistance correlation for completed well. J. Petrol. Sci. Eng..

[27] Borisov J.P. (1964).

[28] Joshi S.D. (1988). Augmentation of well productivity using slant and horizontal wells. J. Petrol. Technol..

[29] Lu J. (1993).

[30] Golan M., Whitson C. (1986).

[31] Hawkins M.F. (1956). A note on the skin effect. Transactions of AIME.

[32] Van Everdingen A.F. (1953). The skin effect and its impediment to fluid flow into a well. Transactions of AIME.

[33] Goode P.A., Thambynayagam R.K.M. (1987). Pressure drawdown and buildup analysis of horizontal wells in anisotropic media. SPE Form. Eval..

[34] Yuan L., Li X., Tan X., Zhang L. (2015). Study on skin factor and productivity of horizontal well after acidizing with non-uniform damage. J. Chem..

[35] Yahui L., Yihan F., Jibin Z., Wei W. (2021). Investigation of productivity prediction method for horizontal wells in gas reservoirs with closed bottom and top boundaries. ACS Omega.

[36] Al-Shammasi A.A. (2001). A review of bubblepoint pressure and oil formation volume factor correlations. SPE Reservoir Eval. Eng..

[37] Elsharkawy A.M., Alikhan A.A. (1997). Correlations for predicting solution gas/oil ratio, oil formation volume factor, and undersaturated oil compressibility. J. Petrol. Sci. Eng..

